# Feasibility, tolerability, and first experience of intracystic treatment with peginterferon alfa-2a in patients with cystic craniopharyngioma

**DOI:** 10.3389/fonc.2024.1401761

**Published:** 2024-07-10

**Authors:** Cora Hedrich, Priya Patel, Lukas Haider, Tracey Taylor, Elaine Lau, Roxanne Hook, Christian Dorfer, Karl Roessler, Natalia Stepien, Maria Aliotti Lippolis, Hannah Schned, Clara Koeller, Lisa Mayr, Amedeo A. Azizi, Andreas Peyrl, Bienvenido Ros Lopez, Alvaro Lassaletta, Julie Bennett, Johannes Gojo, Ute Bartels

**Affiliations:** ^1^ Department of Pediatrics and Adolescent Medicine, Comprehensive Center for Pediatrics and Comprehensive Cancer Center, Medical University of Vienna, Vienna, Austria; ^2^ Department of Pharmacy, The Hospital for Sick Children, Toronto, ON, Canada; ^3^ Department of Biomedical Imaging and Image-Guided Therapy, Medical University of Vienna, Vienna, Austria; ^4^ NMR Research Unit, Queen Square Multiple Sclerosis Centre, Queen Square Institute of Neurology, University College London, London, United Kingdom; ^5^ Department of Neurosurgery, Medical University of Vienna, Vienna, Austria; ^6^ Department of Neurosurgery, Hospital Materno Infantil de Málaga, Málaga, Spain; ^7^ Department of Pediatric Hematology-Oncology, Pediatric Neuro-Oncology Unit, Hospital Infantil Universitario Niño Jesús, Madrid, Spain; ^8^ Department of Radiation Oncology, Clinica Universidad de Navarra, Madrid, Spain; ^9^ Division of Paediatric Haematology and Oncology, Paediatric Brain Tumour Program, The Hospital for Sick Children, Toronto, ON, Canada; ^10^ Division of Medical Oncology and Hematology, Princess Margaret Cancer Centre, Toronto, ON, Canada

**Keywords:** craniopharyngioma, intracystic treatment, peginterferon alfa-2a, pediatric neurooncology, pediatric neurosurgery

## Abstract

**Background:**

Children with craniopharyngiomas (CPs) typically suffer from a life-long chronic disease. The younger the child, the more vulnerable the maturing brain is to invasive therapies such as surgery or radiotherapy. Therefore, treatment modalities facilitating avoidance or delay of invasive therapies are beneficial for these patients. In the last decade, intracystic injection of interferon alfa-2a or alfa-2b evolved as a treatment of choice based on efficacy and minor toxicity. However, the drug is no longer available internationally. After an extensive pharmacological review, peginterferon alfa-2a was identified as the agent with closest similarity.

**Methods:**

A retrospective case series is described, including five patients treated with intracystic peginterferon alfa-2a for cystic CP according to an innovative care protocol. After initial CP cyst aspiration, peginterferon alfa-2a was injected once per week via an Ommaya reservoir for 6 weeks followed by response assessment with MRI.

**Results:**

Patients’ age ranged from 4 to 54 years (four patients <12 years, one adult patient). Intracystic therapy with peginterferon alfa-2a was tolerated well by all five individuals without any major toxicities and resulted in cyst shrinkage in all of the five patients. The importance of a permeability study prior to commencing intracystic therapy became apparent in one patient who suffered from cyst leakage.

**Conclusions:**

Intracystic treatment with peginterferon alfa-2a was found to be a tolerable and efficacious treatment modality in patients with cystic CP. This experience warrants further research with a larger number of patients with measurement of long-term efficacy and safety outcomes.

## Introduction

Adamantinomatous craniopharyngioma (CP) accounts for approximately 5%–10% of pediatric brain tumors. Histologically, they are characterized as benign tumors (1). Gross total resection would represent a cure, but due to spatial proximity or invasion of the tumor to vital anatomical structures, such as the pituitary gland, the optic pathway, the circle of Willis, and the hypothalamus, radical resection could cause serious harm to these structures and, thus, is not generally considered of clinical benefit (2–8). Typical sequelae of surgery include visual impairment, stroke, loss of endocrine function with life-long dependency on hormonal substitution, and life-threatening situations including adrenal crisis or complex electrolyte imbalances and hypothalamic dysfunction, which can result in morbid obesity (3, 4, 8, 9). Another effective method of craniopharyngioma treatment is the use of radiotherapy with good response rates across previous studies (10–13). However, the proximity to the vital structures listed above may cause the same irreversible complications as surgery along with the characteristic long-term complications of radiotherapy, namely, decreased cognitive function, vasculopathies, e.g., Moya Moya, and increased risk of secondary neoplasms (14–20).

Craniopharyngiomas typically consist of solid, calcified, and cystic components (21). Cysts occur in more than 90% of the tumors, often encompassing a major part of the tumor bulk, causing impairment of important structures such as the optic chiasm or obstruction of cerebrospinal fluid circulation (22, 23). This feature is the basis for intracystic therapy, where the neurosurgeon inserts a catheter into the cyst and attaches it to a subgaleal Ommaya reservoir. Via Ommaya reservoir access, drugs can be directly administered into the cyst. In the last decade, interferon alfa-2a and alfa-2b evolved as the treatment of choice due to persuasive efficacy and minor toxicity (22, 24–28).

Interferon alfa-2a and alfa-2b was introduced in the 1950s as an antiviral therapy and has been found to have anti-proliferative activity through inhibition of the JAK/STAT/MAPK pathways and apoptosis through the FAS pathway (29–32).

Typical internationally accepted standard of care schemes such as the São Paulo series or the Toronto protocol consist of the administration of 3 million IU interferon alfa-2a or alfa-2b three times a week (Monday, Wednesday, and Friday) for 4 weeks that were designed as a cycle (24, 33).

Two different forms of interferon alfa, namely, interferon alfa-2a (Roferon^®^-A) and interferon alfa-2b (Intron^®^ A), were previously available but discontinued due to availability of pegylated forms of interferon alfa for the licensed indications. Consequently, the drugs are no longer available.

Being used to the drawbacks of treating rare but very severe diseases with orphan therapies, we set out to find an alternative drug for intracystic therapy to treat children with craniopharyngioma appropriately.

## Material and methods

### Development of an adapted treatment protocol

In brief, a prefilled syringe of 180 mcg (1 ml) peginterferon alfa-2a is injected once weekly via an intracystically placed Ommaya reservoir for 6 weeks (one treatment cycle). At the start of treatment course (day 1), the maximum possible amount of cystic fluid (as patient tolerates) was slowly removed. At the following administrations, the maximal possible amount of fluid (at least 1.5–2 ml) was aspirated. More cycles can be added to obtain a maximal response.

**Figure 1:**
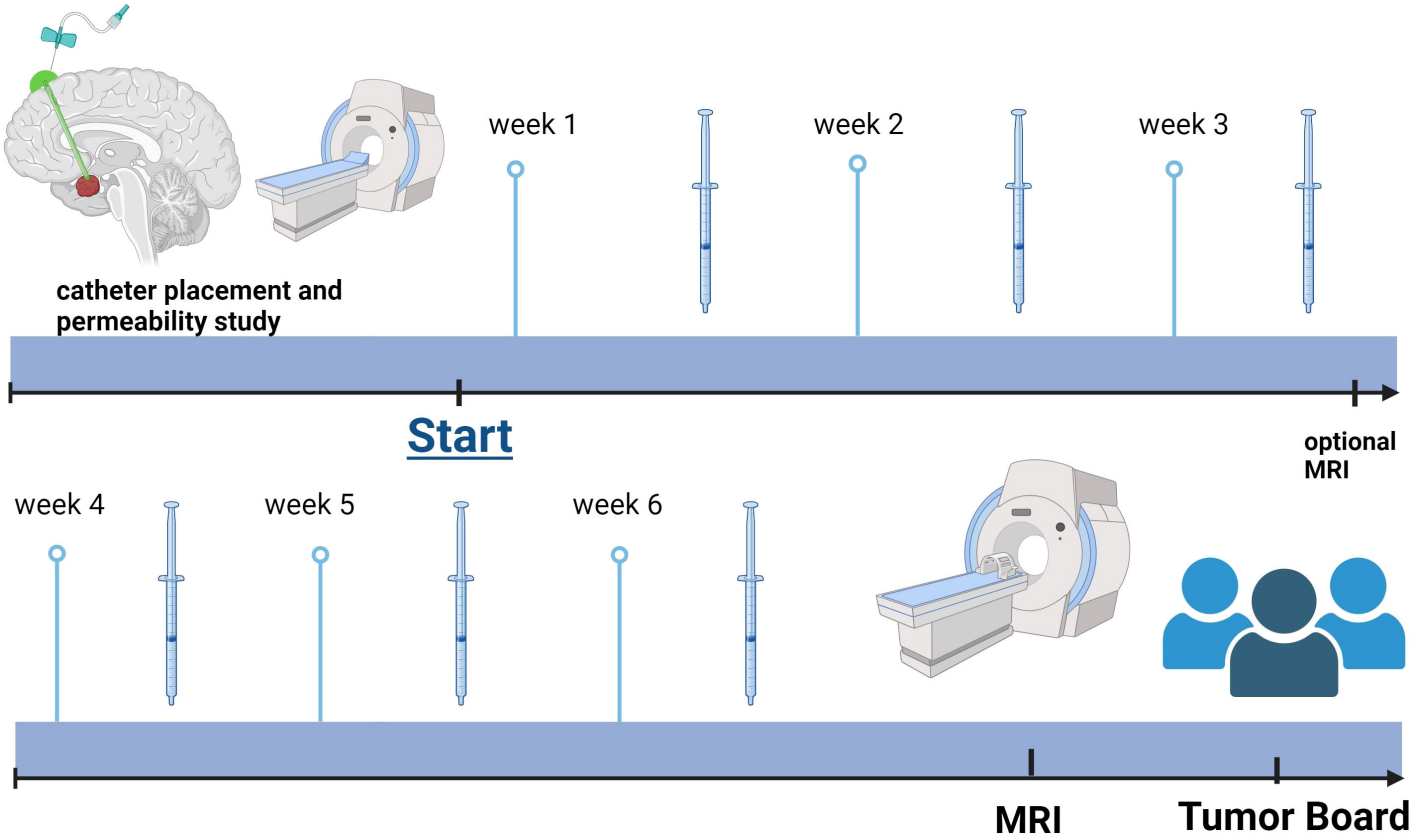
Schematic discription of the treatment protocol.

Depending on the availability of MRI slots in the institution and the need of sedation, fast MRI sequences (see below) every 3 weeks can be performed as optional diagnostic follow-up (**Figure 1**).

### Response assessment

A complete MRI examination (34) for the evaluation of solid tumor components and surrounding key structures was performed at least every 3 months. Notably, a contrast-enhanced MRI is necessary at initial diagnosis or if a solid component progressed, whereas for the evaluation of cyst size, the contrast agent can be dismissed.

The optional fast MRI sequences for response assessment were performed according to the recommendations of the Response Assessment in Pediatric Neuro-Oncology (RAPNO) Working Group and consist of at least three orthogonal T2-weighted sequences and conceivably additional sequences at the radiologist’s discretion for optimal assessment (35). Whenever applicable, cyst size was evaluated by the local radiologist by measurement of cyst size in three dimensions.

Regular neurological exams, vision exams, testing for the pituitary function, and anthropometric measures were performed at least every 3 months for comprehensive evaluation of response assessment.

### Patient data

Five patients from four different institutions (Medical University of Vienna, The Hospital for Sick Children, Princess Margaret Cancer Centre, and Hospital Infantil Universitario Niño Jesús) treated with intracystic peginterferon alfa-2a are included in this report. Data including patient demographics, symptoms at diagnosis or progression, side effects, MRI findings, and response to treatment were collected as standard of care.

In three of the five patients (patient 1, patient 3, and patient 5), the diagnosis of craniopharyngioma was confirmed by histopathology. The other two patients met classical radiological criteria, and the cyst aspiration demonstrated pathognomonic engine oil-like fluid.

### Ethics

Based on thorough reviews of each patients’ case within respective institutional interdisciplinary tumor boards, a suggestion of intracystic treatment was made, and informed consent was given by patient or patient’s legal guardian prior to commencing treatment with peginterferon alfa-2a.

## Results

### Establishment of an alternative for interferon alfa-2a or alfa-2b

A literature search was conducted in October 2020 in Ovid MEDLINE and Embase to determine if there was any published literature with intracystic administration of other interferon products for the treatment of craniopharyngioma. Search terms included interferon and craniopharyngioma, and studies published from 1980 and onwards were included in the search. In addition, a review of all available interferon products was undertaken to better understand the pharmaceutical differences and to make a recommendation for off-label intracystic administration. Based on this review, it was determined that the closest interferon product marketed in Canada to interferon-alfa2 was pegylated interferon (peginterferon)-alfa2a (Pegasys^®^). It was important to note that peginterferon alfa-2a and peginterferon alfa-2b were not interchangeable products as the polyethylene glycol (PEG) chain and bond to the interferon-alfa molecule differed (36). PEGylation is the process of attachment of PEG polymer chains to a molecule and therefore increasing its molecular weight and improving pharmaceutical properties such as the extension of therapy effect. The systemic clearance of peginterferon alfa-2a is approximately 100-fold lower in comparison to interferon alfa-2a. The terminal half-life of peginterferon is approximately 60–80 h after intravenous and 160 h in subcutaneous administration. The dosing that was suggested for peginterferon-alpha2a for craniopharyngioma (one treatment course encompasses once weekly cystic aspirations followed by injection of 180 mcg peginterferon alfa-2a via an Ommaya reservoir for 6 weeks) was extrapolated based on the conversion of subcutaneous hepatitis C dosing of non-pegylated interferon-alfa2a to peginterferon-alpha2a (i.e., peginterferon alfa2a 180 mcg/dose once weekly = interferon alfa2a 3 million units/dose three times per week). The pharmacy team also reviewed the formulation for compatibility with intracystic administration. Typically, formulations administered via an Ommaya reservoir or intrathecally are preservative-free and isotonic. The osmolality of peginterferon alpha-2a is 375–415 mOsmol/kg (slightly hypertonic) (personal communication with Roche, February 2021), and the product also contains benzyl alcohol (37) (preservative), which can cause transient paraplegia or neurotoxicity and polysorbate 80 (38) (solubilizing agent), which can cause allergic reactions. These ingredients would not typically make for an ideal formulation for intracystic administration. However, both of these ingredients are also found in interferon-alfa2a (39), which has been used intracystically across multiple patient series. Thus, we decided to offer intracystic administration with the peginterferon-alfa-2a product disclosing the potential risks to the patient and family. First experiences in five patients treated in exact accordance to the innovative care protocol at four different institutions are presented in this article.

### First clinical case series

**Figure 2:**
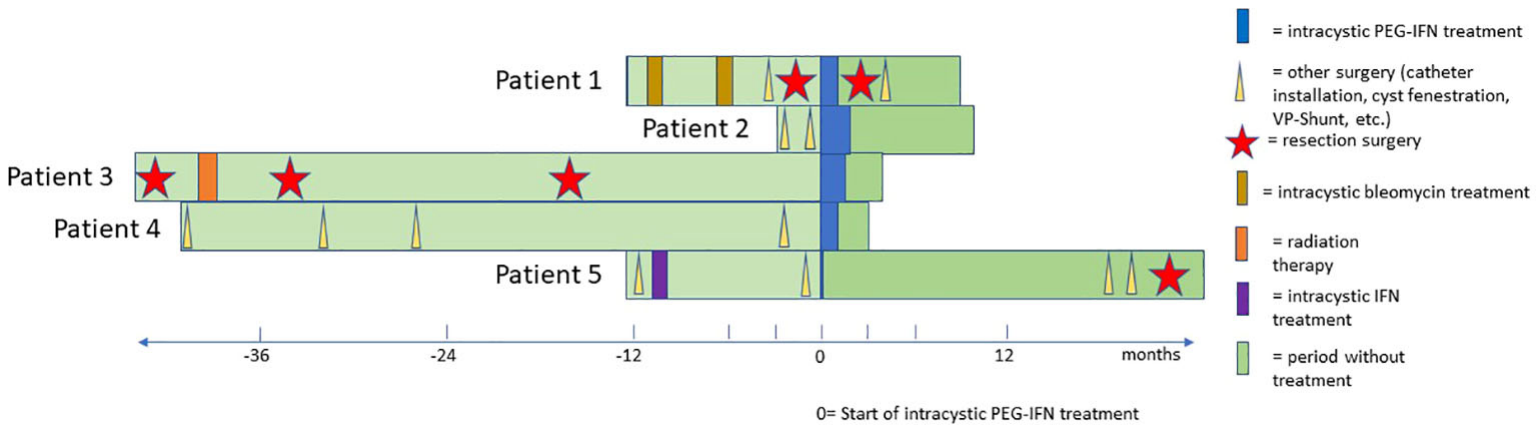
Swimmer’s plot that shows the individual treatment course of each patient from the initial diagnosis until the preparation of the manuscript. The vertical purple line represents the start of cystic Peg Interferon alfa treatment in each patient.

**Figure 3:**
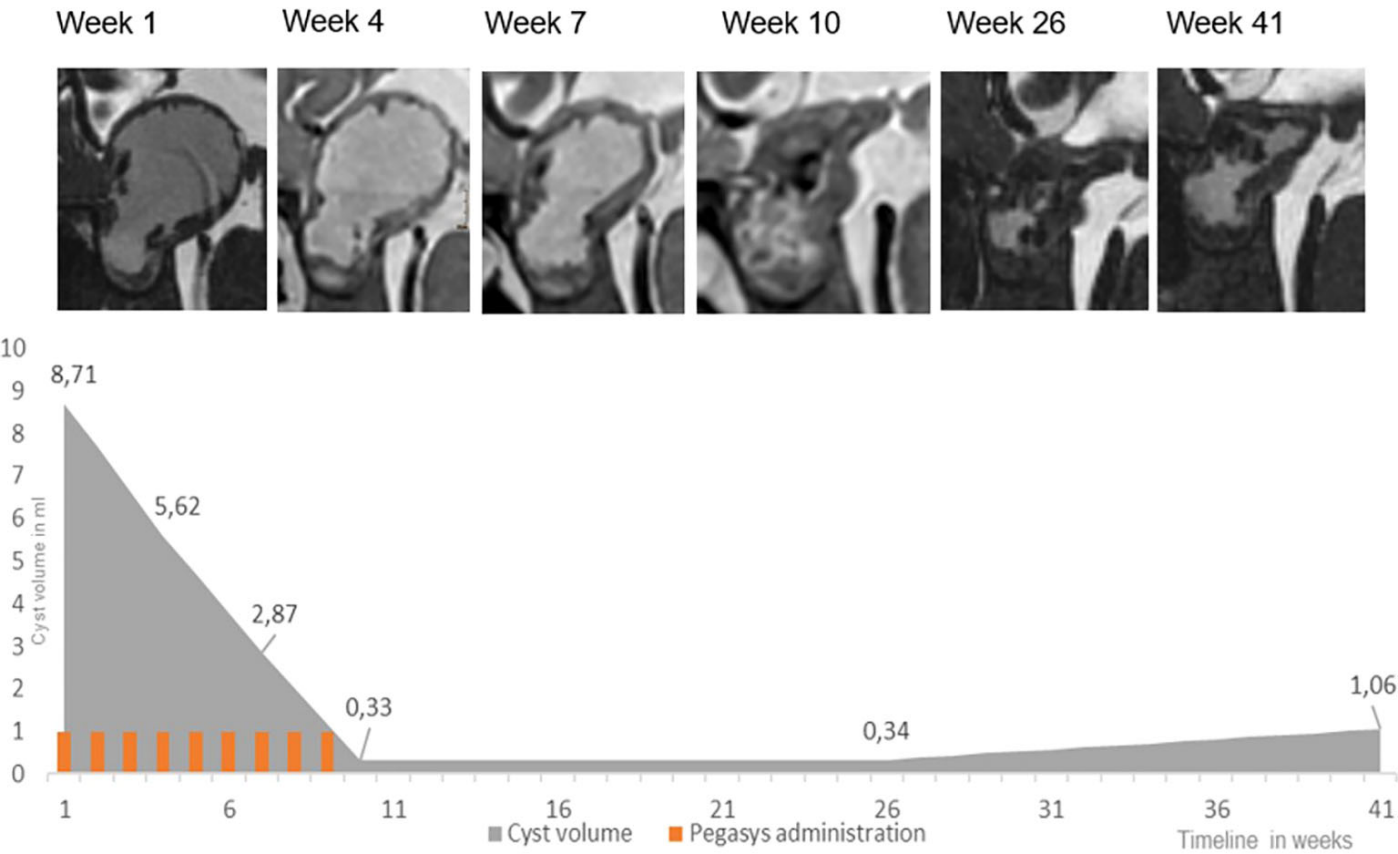
Peginterferon alfa-2a treatment in patient 2: high resolution, isotropic, strongly T2-weighted sequences, with 0.7 mm voxel size were acquired at 1.5 T using a Siemens Aera (CISS) and Philips Ingenia (BTFE). The lower panel indicates development of the cyst volume and treatment.

Within an international collaboration, we compiled the first five cases treated with peginterferon alfa-2a (**Table 1**; **Figures 2**, **3**) according to the same innovative care protocol for treatment guidance (**Supplementary Material 1**).

**Table 1:** * 0= stable disease or mixed response, 1= cyst shrinkage, 2= progression during therapy; **collapse of central multiloculated cyst but growth of small peripheral isolated cysts; ***0= no relevant side effects, Common Terminology Criteria for Adverse Events (CTCAE) Grade.

	Age (years)	Sex	Prior therapy	Type	Number of PEG-IFN applications	Response*	Cyst volume before/after PEG-IFN treatment	Clinical benefit	Side effects***
Patient 1^1^	7	m	2 cycles bleomycine	Multicystic	6	0	5.8 ml/collapse**	Improvement of headaches during treatment	0
Patient 2^2^	4	f	1x Cyst drainage	Monocystic	9	1	8.71 ml/0.33 ml	Minimal improvement of vision	0
Patient 3^3^	54	m	3x resection Radiation therapy	Multicystic	6	1	9.10 ml/2.10 ml	improvement of headaches, vision, and neurocognition	0
Patient 4^4^	5	f	1x Cyst drainage	Multicystic	6	1	4 ml/0.5 ml	Improvement of headaches, preservation of vision	CTCAE grade 1 (fatigue)
Patient 5^2^	12	m	1x resection	Multicystic	1	1	N/A	Preservation of vision	CTCAE grade 3 hypersensitivity reaction (hospitalization)

1: Regional University Hospital, Malaga, Spain;

2: Medical University of Vienna, Austria;

3: Princess Margaret Hospital, Toronto, Canada,

4: Hospital for Sick Children, Toronto, Canada.

Patient 1 was a 7-year old boy with a CP who was previously treated with intracystic bleomycin consisting of two cycles in an interval of 6 months. Subsequently, a VP-shunt insertion and a pterional partial resection took place. Due to further cystic progression only 2 months later, he received intracystic peginterferon alfa-2a, which he tolerated well without any side effects. As a response to treatment, the central multiloculated cyst collapsed, but some small peripheral isolated cysts progressed.

Patient 2 was a 4-year-old patient who was newly diagnosed with a monocystic craniopharyngioma. A catheter was inserted to allow for aspiration of cyst fluid without the instillation of medication. This therapeutic effect was only short-lived, as the cyst returned to its original size after a month. In addition, visual acuity decreased during this interval (**Table 1**). At that time, no cyst fluid could be aspirated, leading to a catheter revision. Subsequently, treatment with intracystic peginterferon alfa-2a was given. The patient tolerated the therapy well, and only reported local skin pain in the area of the Ommaya reservoir and headache during the aspiration of the cyst. The residual cyst volume continuously decreased during treatment and shrunk to such a low volume, that we considered the therapy successfully completed after 9 injections (**Figure 1**). Four weeks after the first administration of peginterferon alfa-2a, the vision improved to a small extent and remained stable at last follow-up. The CP cyst remained stable in size for 41 weeks after the completion of the peginteferon alfa-2a therapy (**Figure 3**).

Patient 3 is a 54-year-old man who was histologically diagnosed with adamantinomatous CP. Initially, he underwent a tumor resection followed by 54 Gy in 30 fractions radiation therapy. He suffered from a local recurrence twice and received debulking surgery at 1 and 3 years after the initial diagnosis. The patient suffered a stroke after the third surgery, which led to right-sided paralysis, and he experienced another cystic progression within the same year. Despite cyst aspirations, the patient had nightly headaches and visual disturbance and demonstrated difficulties with memory and slow speech due to fast refilling of the cyst. Hence, a course of intracystic peginteferon alfa-2a was suggested. After the second injection, he experienced nausea. There were no further toxicities, and he tolerated all the other procedures well. Due to a vacation, he interrupted the therapy for 2 weeks during the 6-week cycle. He subjectively noted an improvement of the headaches and the vision. Neuropsychological testing showed amelioration of memory and speech function. The assessment at the treating institution reported a decrease in the cyst volume from 9.1 ml to 2.1 ml (total shrinkage of 77%). One month after completion of treatment, the MRI scan showed a re-accumulation of fluid in the cyst. The ophthalmic examination showed a significant worsening of the vision (right eye, 20/25; left eye, 20/200). He had temporary relief of symptoms with a cyst aspiration, and another course of intracystic peginterferon alfa-2a therapy has been initiated for this patient. In this patient, some clinical improvement may be attributed to the cyst aspirations alone. However, the interval between the interventions and relapse increased after peginterferon application.

Patient 4 is a 5-year-old girl who was treated with cyst drainage and later catheter insertion and catheter revision at the first months of diagnosis of her multicystic craniopharyngioma. When a new posterior cyst showed a progression after 3 years, a new catheter was inserted medial to the pre-existing frontal one. The patient started treatment with peginterferon alfa-2a 3 years after the initial diagnosis and received one cycle. Prior to treatment, the patient reported multiple severe episodes of headaches daily. In the course of the treatment, they improved significantly and decreased in frequency to only one to two times per week. During the treatment, the patient had some flu-like symptoms and fatigue. Noteworthy, the patient did not take her prescribed hydrocortisone substitution for several days due to the taste of the pill, which may have contributed to her fatigue.

Patient 5 was a 12-year-old boy who had previously received treatment with intracystic Interferon alfa-2a at initial diagnosis (three times per week for 4 weeks) with good response. One year later, the patient suffered cystic progression, and due to catheter dysfunction, an operative revision was performed to facilitate intracystic treatment. Two days after the first administration of peginteferon alfa-2a, the patient experienced a distinct systemic reaction with general skin rash and edema. The patient was immediately admitted to his local hospital where he was treated with corticosteroids and antihistamines and showed fast remission within hours. The intracystic therapy was discontinued in this patient. Retrospectively, the catheter was dislocated outside of the cyst due to intraoperative collapse resulting in the administration of the substance in the adjacent tissue inducing a systemic reaction. Subsequently, the CP remained stable for 19 months before a new cyst emerged again, necessitating surgical treatment and placement of a new catheter. This time, a permeability study was performed, indicating leakage of contrast media. Consequently, no intracystic treatment was administered, and a partial resection followed by proton beam therapy was performed.

## Discussion

Intracystic therapy with interferon alfa-2a has been used as an established modality in several institutions in the therapy of children with CP. Analysis of previous retrospective studies demonstrated an advantage in effectiveness and tolerability for intracystic interferon compared to other established therapies (22, 24–26, 31, 33). The largest trial on intratumoral interferon alfa-2a was published by the São Paulo team, who are also the pioneers in the development of intratumoral Interferon alfa-2a treatment (24). Clinical and radiological improvement was achieved in 76% of the 60 patients included (25). However, this review has no sufficient long-term follow-up data yet to inform on the duration of the cyst reduction and the time to subsequent progression.

A global, multicenter assessment on behalf of SIOPE and ISPN represents another broad clinical experience on intracystic therapy with Interferon-alfa including 56 children in this retrospective study (26). While treatment with the previously used (unpegylated) intracystic interferon had shown to delay the need for surgical resection or radiotherapy for a median time of 5.8 years, it is important to note that the long-term efficacy of treatment with intracystic peginterferon still needs to be proven within future clinical trials. The authors proposed a global, prospective randomized clinical trial of intracystic interferon in childhood CP, but the randomized trial concept was considered too expensive and prone to fail in this rare disease by funding resources. Hence, this case series fills an important gap and demonstrates the feasibility of an intracystic peginterferon alfa-2a regimen. It is relevant to address several limitations inherent in this study. First, the series includes only five patients. However, since craniopharyngioma is a rare disease and not all craniopharyngioma patients are suitable for intracystic treatment, we consider our experience of high importance to the neuro-oncology community. Second, the data exhibit a certain heterogeneity such as differences in the decision process on the indication for the treatment, the selection of suitable patients, the previous treatments of the patients, and the follow-up due to the nature of a retrospective study. Additionally, this report does not inform on the neurosurgical aspects to fulfill the requirement of a functional intracystic catheter, which may also limit applicability of intracystic treatment. Case 5 demonstrated the importance of pursuing a permeability study at least 2 weeks postoperatively and prior to the start of intracystic treatment to rule out leakage, most likely the cause of patient’s systemic toxicities (40). One case report of a 13-year-old patient treated with intracystic plus concomitantly subcutaneous pegylated interferon alfa-2b (41) described irreversible visual field loss and confirmed leakage from the intracystic catheter via computed tomographic imaging as the cause.

Herein, we present the first series of five patients treated with intracystic peginterferon alfa-2a. All patients showed prior progressive disease according to the new consensus guidelines of the Response Assessment in Pediatric Neuro-Oncology (RAPNO) committee indicated by change of cyst size and deterioration of a clinical parameter such as a new functional impairment or the need of surgical intervention (35). In general, intracystic treatment is considered particularly effective in monocystic CP. In multicystic CP, peripherical cysts may not communicate with the drained main cyst and are therefore not accessible to the intracystic treatment. This was demonstrated in patient 1 in whom the central multiloculated cyst collapsed, but a growth of small peripherical cysts was noted at the end of the treatment. A proactive approach towards intracystic therapy may postpone morbidities associated with surgical resection and irradiation as the insertion of a catheter is a low-risk operation (9), and there is a certain risk that newly developed functional impairments are not reversible once they occur. There is no doubt that delaying more aggressive surgical interventions and/or radiotherapy will be of significant benefit to young children and their maturing brains. This is in line with the main goal in treatment of CP as a chronic disease with a paradigm to maintain good quality of life (QoL) with minimally invasive intervention given the potential for substantial morbidity in the long-term outcomes of CP patients (21, 42–44).

While our findings prove the feasibility of intracystic peginterferon alfa-2a, no definite conclusions on the future significance can be made. To address these gaps, collaborative efforts across a large number of brain tumor centers must be made to design clinical trials with adherence to well-defined entry criteria, standardized treatment protocols, and interpretation of results by a reference center. With the recent advances in precision medicine, some potential targets such as IL-6, PD1/PD-L1, MEK, IDO-1, and others have been identified for the treatment of craniopharyngioma (45–47). For example, the CONNECT 1905 phase 2 study analyzes the effects in craniopharyngioma patients treated with systemic Tocilizumab, an IL-6 receptor antagonist that is approved for the treatment of arthritis. Additionally, bevacizumab has been shown to effectively reduce cyst size in selected cases (48). The local installation of medication as with intracystic peginteferon alfa-2a appears as an attractive alternative to avoid systemic, potentially persisting side effects, in young children.

An important advantage of the pegylated formula is the possibility of a weekly administration, which represents less applications compared to the previous non-pegylated interferon therapy. This factor represents an essential benefit for the child and its caregivers, as fewer procedures are required. The administration can be done in an outpatient setting. As a consequence, the child has fewer hospital visits and less interruptions in everyday life for a whole family. Lastly, if response is present but insufficient, additional cycles can be added as needed.

## Conclusion

In this case series experience using peginterferon alfa-2a for intracystic treatment, its feasibility, tolerability, and response measured in cyst shrinkage were demonstrated. The results of this case series are encouraging that peginterferon could replace previous interferon formulations with the added benefit of less frequent administrations.

## Data availability statement

The raw data supporting the conclusions of this article will be made available by the authors, without undue reservation.

## Ethics statement

Local ethical approval and informed consent was available at the respective institutions in accordance with the local legislation and institutional requirements. Written informed consent was obtained from the individual(s), and minor(s)’ legal guardian/next of kin, for the publication of any potentially identifiable images or data included in this article.

## Author contributions

CH: Conceptualization, Data curation, Formal analysis, Investigation, Methodology, Project administration, Software, Validation, Visualization, Writing – original draft, Writing – review & editing. PP: Conceptualization, Investigation, Writing – original draft, Writing – review & editing. LH: Writing – original draft, Writing – review & editing, Conceptualization, Data curation, Formal analysis, Investigation, Methodology, Project administration, Resources, Software, Validation, Visualization. TT: Conceptualization, Investigation, Writing – review & editing. EL: Conceptualization, Investigation, Validation, Writing – review & editing. RH: Conceptualization, Investigation, Writing – review & editing. CD: Conceptualization, Formal analysis, Methodology, Validation, Writing – review & editing. KR: Validation, Writing – review & editing. NS: Data curation, Investigation, Software, Validation, Visualization, Writing – review & editing. ML: Data curation, Investigation, Visualization, Writing – review & editing. HS: Data curation, Investigation, Project administration, Writing – review & editing. CK: Data curation, Investigation, Writing – review & editing. LM: Data curation, Investigation, Methodology, Software, Validation, Visualization, Writing – review & editing. AA: Data curation, Investigation, Methodology, Validation, Writing – review & editing. AP: Data curation, Investigation, Methodology, Validation, Writing – review & editing. BR: Data curation, Investigation, Writing – review & editing. AL: Data curation, Investigation, Writing – review & editing. JB: Data curation, Investigation, Writing – review & editing. JG: Writing – original draft, Writing – review & editing, Conceptualization, Data curation, Formal analysis, Funding acquisition, Investigation, Methodology, Project administration, Resources, Software, Supervision, Validation, Visualization. UB: Conceptualization, Data curation, Formal analysis, Investigation, Methodology, Project administration, Resources, Supervision, Validation, Writing – original draft, Writing – review & editing.
