# Supplementary material for: Feasibility, tolerability, and first experience of intracystic treatment with peginterferon alfa-2a in patients with cystic craniopharyngioma
Source: Front Oncol. 2024 Jul 10;14:1401761. doi: 10.3389/fonc.2024.1401761 (PMC11266088; doi:10.3389/fonc.2024.1401761)
Supplement: Supplementary file 1 [file DataSheet_1.pdf]

MEDICAL UNIVERSITY OF VIENNA

SickKids® THE HOSPITAL FOR SICK CHILDREN

Intracystic treatment with Peginterferon alfa-2a in patients with cystic craniopharyngioma: the protocol

Cora Hedrich[1Ω], Priya Patel[2Ω], Lukas Haider[3, 4], Tracey Taylor[2], Elaine Lau[2], Roxanne Hook[2], Christian Dorfer[5], Karl Roessler[5], Natalia Stepien[1], Maria Aliotti Lippolis[1], Hannah Schned[1], Clara Koeller[1], Lisa Mayr[1], Amedeo A. Azizi[1], Andreas Peyrl[1], Bienvenido Ros Lopez[6], Alvaro Lassaletta[7,8], Julie Bennett[9,10] Johannes Gojo[1Σ], Ute Bartels[9Σ]

Affiliations

[1] Department of Pediatrics and Adolescent Medicine, Comprehensive Center for Pediatrics and Comprehensive Cancer Center, Medical University of Vienna, Vienna, Austria.

[2] Department of Pharmacy, The Hospital for Sick Children, Toronto, Ontario, Canada [3] Department of Biomedical Imaging and Image-Guided Therapy, Medical University of Vienna, Vienna, Austria.

[4] NMR Research Unit, Queen Square Multiple Sclerosis Centre, Queen Square Institute of Neurology, University College London, London, UK

[5] Department of Neurosurgery, Medical University of Vienna, Vienna, Austria.

[6] Department of Neurosurgery, Hospital Materno Infantil de Málaga, Málaga, Spain

[7] Department of Pediatric Hematology-Oncology, Pediatric Neuro-Oncology Unit. Hospital Infantil Universitario Niño Jesús, Madrid, Spain

[8] Department of Radiation Oncology. Clinica Universidad de Navarra. Madrid. Spain

[9] Division of Paediatric Haematology and Oncology, Paediatric Brain Tumour Program, The Hospital for Sick Children, Toronto, Ontario, Canada [10] Division of Medical Oncology and Hematology, Princess Margaret Cancer Centre, Toronto, Ontario, Canada

Ω Co-first author ∑ Co-senior author

Drug	Route	Dosage	Schedule
**Peginterferon alfa-2a** (prefilled Syringe of 180 mcg Pegasys®)	Intracystic injection	180 mcg	1x administration every 7 days in week 1-6
1 cycle= 6 weeks treatment<br>MRI scan after 6 weeks and (if possible) every 3 weeks (fast sequences sufficient for the measurement of the cyst size)<br>Cycle may be shortened or repeated depending on response			
No dosage modificiations are suggested			

Materials

- 1x prefilled Syringe of 180 mcg Pegasys®
- Disposable razor, soap, kidney dish
- Surgical face mask for every person attending
- Nonsterile working pad
- Sterile surgical sheet
- 1x sterile surgical gloves
- 1x sterile chemotherapy gloves (PPE)
- Tinted desinfection agent (Isozid®)
- Untinted desinfection agent (Isozid®)
- 1 package with 10 sterile gauze compresses 7,5x 7,5cm
- 2 Butterfly needles (25G)
- 3 packages with sterile pads 5x5cm
- 2 tubes
- 2 cannulas
- 1 package with sterile pads 5x5 cm
- 5 ml Liquor cerebrospinalis (artificial CSF)
- 3x 5ml syringes
- Yellow sharp box
- Sterile Patch 4x5 cm
- Tube, e.g. Vacuette®

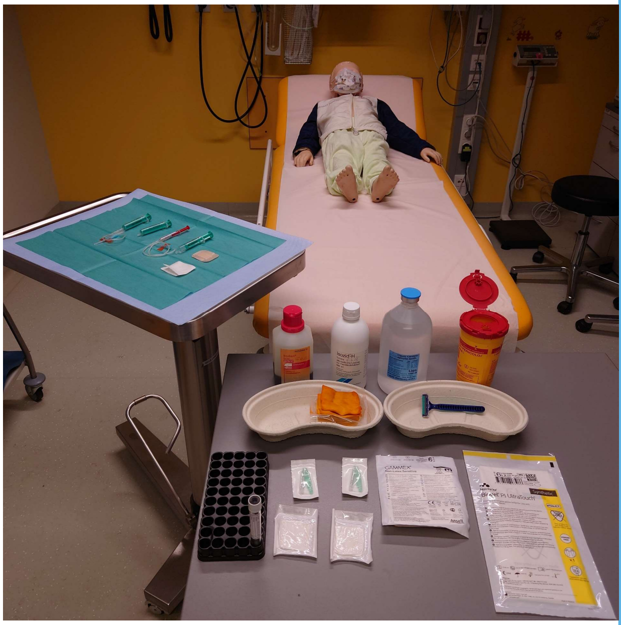

Requirements for the intracystic therapy via an Ommaya reservoir

- No sign of cyst leak in the permeability study performed at least two weeks after the insertion of the catheter and approval of the neurosurgeon
- No sign of CNS infection
- Thrombocyte count >30 000/ul
- Patients with hypocortisolism might take a stress dose of their substitution

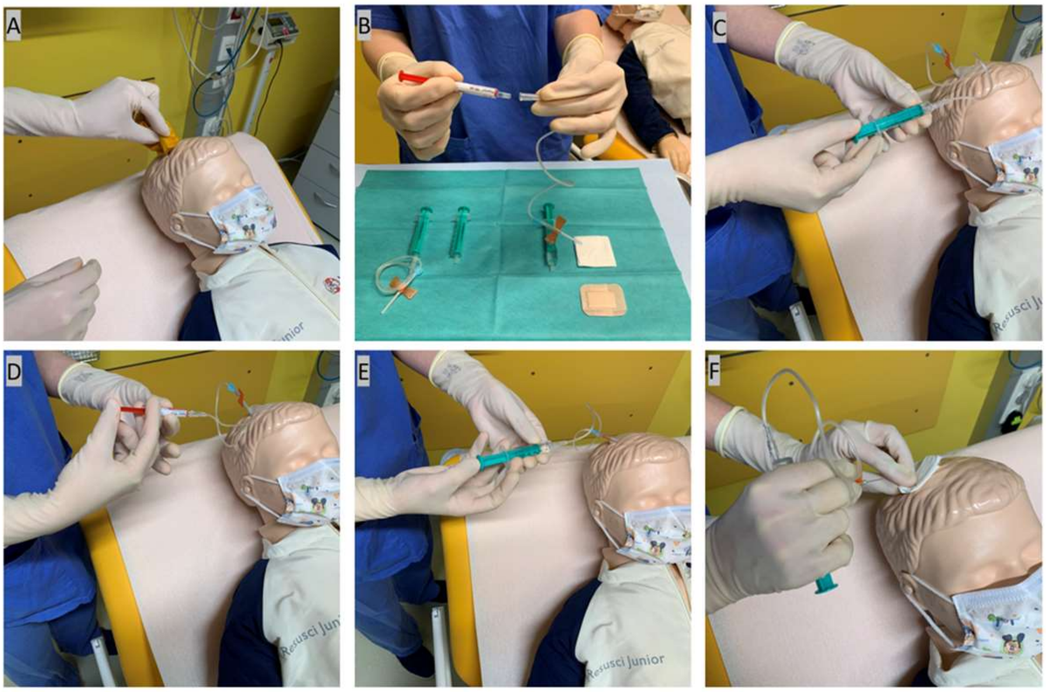

Procedure

- The treatment is performed by a pediatric neurooncologist or neurosurgeon and a trained nurse in a treatment room under mainly sterile conditions. Every person in the room must wear a face mask. The door remains closed during the treatment and the procedure must not be disrupted from the outside.
- To ensure adequate neurological assessment during the procedure sedation should be avoided if it is possible to perform it under safe conditions depending on the child's cooperation. In small children an additional nurse or clinical psychologist is recommended to distract the child and guide it through the procedure by talking or making puns. Audioplayers or phones can be used to play music or videos to help the child to relax.
- The patient is positioned in a semi-recumbent position (approx. 30-45° inclination of the head part of the treatment table). The hair over the Ommaya reservoir is removed with a disposable wet razor.
- Thorough skin disinfection (10x) with sterile gloves and tinted Isozid® by the doctor (Figure 1, A)
- Change of gloves to sterile chemotherapy gloves and preparation of the utensils on the sterile surgical sheet by sterile handing out by the nurse: 2 butterfly needles, 3x 5ml syringes, artificial CSF, cannula, sterile pad, sterile plaster. The non-sterile Pegasys® syringe is taken with a Isozid® soaked pad without touching the sterile gloves and desinfected thoroughly. A four-eye control (identity of the patient, correct medication, correct day of application, expiration date of the mediacation) is carried out between the doctor and the nurse.
- A 5 ml syringe is connected to the first butterfly needle. The Pegasys® syringe is connected to the second butterfly needle and the line is pre-rinsed with the medication. (Figure 1, B)
- The doctor punctures the Ommaya reservoir with the first butterfly needle and aspires the cyst fluid slowly. (Figure 1, C) At start of treatment course (day 1) the maximum possible amount of cystic fluid (as patient tolerates) should be slowly removed. At the following administration at least 1.5- 2 ml should be aspired.
- The nurse takes over the syringe and divides the cyst fluid into the previously disinfected Vacuette for a microbiology culture and Cryotubes for Biomarker studies.
- The physician punctures the Ommaya reservoir with the second butterfly needle that is connected to the Pegasys® syringe and applies the drug. (Figure 1, D) Then the Ommaya reservoir is rinsed with a defined amount of artificial CSF (calculation see below). (Figure 1, E)
- After removal of the needle the puncture site is compressed gently with a dry and sterile pad and then sticked with a sterile patch. (Figure 1, F)
- After the procedure the child is observed in a patient room for approximately 15 minutes depending on the tolerability of the previous injections. After the first application the patient is observed for 24 hours in the ward.
- Correct documentation of the application in the patient's chart and medical report.

Comments

- General definition of amount of flush volume with artificial CSF
  -) Small Ommaya reservoir (size 1.5 cm, flushing volume 0.31 ml): rinse with 1.5 ml artificial CSF
  -) Large Ommaya reservoir (size 2.5 cm, flushing volume 1.14 ml): rinse with 2 ml artificial CSF.
  The amount of flush volume is adapted to the aspirable amount of cyst fluid and the clinical condition of the child (e.g. pain, visual symptoms). If no fluid is aspirable, no or very little artificial CSF should be flushed. If it is known that several millilitres are aspirable in a patient, the administration of Pegasys® can be done by connecting the syringe to the first butterfly needle abstaining from a second puncture.
